# Priorities for addressing substance use disorder in humanitarian settings

**DOI:** 10.1186/s13031-021-00407-z

**Published:** 2021-09-23

**Authors:** M. Claire Greene, Stephanie Haddad, Anja Busse, Nadine Ezard, Peter Ventevogel, Lina Demis, Sachi Inoue, Jan-Christopher Gumm, Giovanna Campello, Wietse A. Tol, Jeremy C. Kane

**Affiliations:** 1 Prevention, Treatment and Rehabilitation Section, United Nations Office on Drugs and Crime, Vienna International Centre, Wagramer Strasse 5, 1400 Vienna, Austria; 2 St Vincent’s Hospital Sydney/National Centre for Clinical Research in Emerging Drugs/NDARC UNSW, 390 Victoria Street, Darlinghurst, NSW Australia; 3 Public Health Section, United Nations High Commissioner for Refugees, Rue de Montbrillant 94, 1201 Geneve, Switzerland; 4 Department of Epidemiology, Columbia University Mailman School of Public Health, 722 W 168th Street, New York, NY USA; 5 Department of Public Health, University of Copenhagen, Nørregade 10, 1165 Copenhagen, Denmark; 6 Program on Forced Migration and Health, Heilbrunn Department of Population and Family Health, Columbia University Mailman School of Public Health, 60 Haven Avenue, New York, NY 10032 USA

**Keywords:** Substance use, Alcohol, Drugs, Humanitarian settings

## Abstract

**Background:**

Populations affected by humanitarian emergencies are vulnerable to substance (alcohol and other drug) use disorders, yet treatment and prevention services are scarce. Delivering substance use disorder treatment services in humanitarian settings is hampered by limited guidance around the preparation, implementation, and evaluation of substance use disorder treatment programs. This study aims to identify and prioritize key gaps and opportunities for addressing substance use disorder in humanitarian settings.

**Methods:**

UNODC convened a consultation meeting (n = 110) in coordination with UNHCR and WHO and administered an online survey (n = 34) to, thirteen program administrators and policymakers, eleven service providers, nine researchers, and one person with lived experience to explore best practices and challenges to addressing substance use disorder in diverse populations and contexts. Participants presented best practices for addressing substance use disorder, identified and ranked challenges and opportunities for improving the delivery of substance use disorder treatment interventions, and provided recommendations for guidelines that would facilitate the delivery of substance use disorder treatment services in humanitarian emergencies.

**Results:**

Participants agreed on key principles for delivering substance use disorder treatment in humanitarian settings that centered on community engagement and building trust, integrated service delivery models, reducing stigma, considering culture and context in service delivery, and an ethical ‘do no harm’ approach. Specific gaps in knowledge that precluded the delivery of appropriate substance use disorder treatment include limited knowledge of the burden and patterns of substance use in humanitarian settings, the effectiveness of substance use disorder treatment services in humanitarian settings, and strategies for adapting and implementing interventions for a given population and humanitarian context. Participants emphasized the need to strengthen awareness and commitment related to the burden of substance use disorder treatment interventions among communities, practitioners, and policymakers in humanitarian settings.

**Conclusions:**

Results from this consultation process highlight existing gaps in knowledge related to the epidemiology and treatment of substance use disorders in humanitarian emergencies. Epidemiological, intervention, and implementation research as well as operational guidance are needed to fill these gaps and improve access to substance use treatment services in humanitarian settings.

**Supplementary Information:**

The online version contains supplementary material available at 10.1186/s13031-021-00407-z.

## Background

Global displacement reached unprecedented levels in 2021 with over 80 million people forced to flee their homes due to humanitarian emergencies [1]. Trends in humanitarian emergencies, defined as an event or series of events that disrupt and threaten the safety, health, and wellbeing of population [2, 3], have shifted in recent years. Political conflicts have become more intense, more people have been internally displaced by conflict and violence in the last decade than ever before, hunger and poverty have increased, and climate change has precipitated natural disasters and severe weather events, all of which have been compounded by the direct and indirect consequences of the COVID-19 pandemic [4]. Humanitarian emergencies have become increasingly characterized by the confluence of these hazards and threats to population wellbeing, which exacerbate their respective impacts on morbidity and mortality [3].

Populations affected by humanitarian emergencies may experience an elevated vulnerability to alcohol and other drug use disorders, hereafter referred to as ‘substance use disorders’. While there is an absence of prospective longitudinal studies examining changes in risk and incidence of substance use disorder among populations affected by humanitarian emergencies, qualitative and quantitative cross-sectional or retrospective studies suggest that exposure to humanitarian emergencies is associated with substance use disorder and related harms. For example, a systematic review of opiate use in the context of armed conflict found that five out of six studies suggested that opiate use and related harms, including hospital admissions and drug-related deaths, increased after periods of armed conflict [5]. Elevated risk for substance use disorder in these settings may be conferred through exposure to potentially traumatic events, adversity and stress, and the higher prevalence of mental health problems that commonly co-occur with substance use disorders [6–11], as well as increased exposure to readily available substances and potential breakdown of social norms around substance use. Substance use disorder has been described in the context of humanitarian emergencies [7, 12–16]. In studies using validated measures, estimates of the prevalence of alcohol dependence has ranged from < 1–42%, and estimates of drug dependence, type not specified, has ranged from 1 to 20% in populations displaced by emergencies [11]. A limitation of existing literature is that most studies do not provide information about the types of drugs used among those with drug dependence [11].

While the mental health and substance use disorder-related needs of those in humanitarian settings are high, resources are scarce and the existing health infrastructure is often too overwhelmed to provide sufficient care to all those who need it [17, 18]. Additionally, the resources needed to address substance use disorder in humanitarian settings depends on the type(s) of substances that are prevalent (e.g. alcohol and/or different types of drugs), the patterns of use, and the populations that may be most at risk, all of which vary across settings. An analysis of consultations in 90 refugee settings documented notable differences in contact coverage with primary care for mental, neurological, and substance use (MNS) problems, with particularly low contact coverage for alcohol and other drug use disorders [19]. Although this may be due, in part, to the varying prevalence of MNS problems in different populations, other resource and access constraints are likely to contribute to this lack of coverage such as limited training and capacity, access to care, availability of medications, and norms around help-seeking for substance use disorders are likely to also explain the low treated prevalence of substance use disorders [19–21]. Other challenges for implementation of substance use disorder treatment programs in humanitarian settings include low political prioritization, lack of coordination and integration of substance use and related services, competing priorities, high staff turnover, structural and community stigma, and logistical challenges [21–23].

Although interventions and guidelines have been developed to address substance disorders in humanitarian settings [24–27], there is little research on their effectiveness and uptake. Interventions that have been recommended to address harmful alcohol and drug use in these settings include brief motivational conversation, self-help groups, stress management, social support strengthening, and withdrawal management [26]. Many such interventions are derived from evidence in high-resource and non-humanitarian contexts and their relevance, safety, feasibility, and effectiveness in humanitarian settings has not yet been discerned. The dearth in literature reflects current research and funding priorities and, subsequently, the programming capacities of humanitarian aid organizations. The limited research on these interventions in humanitarian settings restricts the capacity of practitioners to develop effective implementation strategies. Moreover, the limited evidence available is a challenge for policy and high-level decision makers with the responsibility to address the substance use disorder related needs of populations experiencing emergencies and displacement.

Calls to expand the knowledge base for substance use disorder interventions, reflect humanitarian programming capacities and practitioners’ need for additional guidance to inform planning and implementation for these programs [28–31]. The Prevention, Rehabilitation, and Treatment Section of the United Nations Office on Drugs and Crime (UNODC), whose mandate is to develop and disseminate best practices in substance use disorder epidemiology, prevention, and treatment, has established an initiative to address substance use disorder and associated health and social consequences in humanitarian settings. The goal of this initiative is to increase access to substance use disorder treatment in humanitarian settings by generating practical resources to support program planners and practitioners in substance use disorder treatment delivery. In effort to guide the development of a technical handbook to achieve this goal, this study aimed to explore the major gaps, challenges, and priorities for addressing substance use in humanitarian emergencies.

## Methods

In September 2020, UNODC in coordination with the United Nations High Commissioner for Refugees (UNHCR) and the World Health Organization (WHO), convened a three-day expert consultation meeting. Participants were invited by stakeholders to participate based on their role as a policymaker, practitioner, advocate, researcher, or person with lived experience working on issues related to substance use interventions in humanitarian settings. United Nations Member States were invited to nominate a representative to attend the meeting and other experts were identified through referral and the networks of UNODC and other meeting organizers. We also invited authors of relevant publications that were identified through literature review. No additional eligibility criteria were set for participation. The meeting included presentations on best practices, experiences, and research from 55 participants. Plenary discussion and small breakout groups were used to identify key populations, gaps in knowledge, principles, and resources needed to improve the treatment of substance use disorder in humanitarian emergencies. One member of our team took detailed notes on each presentation and discussion, which were also recorded. We collected all presentation materials from participants for the analysis.

In February 2021, all expert consultation meeting attendees who were not directly involved in the development of the technical handbook (n = 92) were invited to participate in a 20-min online survey (see Additional File 1). Of the 92 expert meeting attendees who were invited to participate, 34 completed the survey. The objectives of the survey were to obtain specific recommendations on: (1) essential services to address substance use disorders in humanitarian settings; (2) key gaps in knowledge and skills needed to treat substance use disorders in humanitarian settings; (3) challenges for delivering substance use interventions in humanitarian settings; (4) priorities for improving the implementation and delivery of substance use interventions in humanitarian settings; and (5) policy recommendations. We drew from recommendations for designing questions for surveys as part of the Delphi method, which resulted in questions with open-ended response formats as well as ranking tasks where respondents were asked to prioritize a list of services, needs/gaps, resources, and challenges [32, 33]. Survey questions were developed by study investigators and were informed by the themes that emerged from the expert meeting as well as the information needed to develop the technical handbook. At the end of the survey participants were invited to recommend other experts to invite to complete the survey. The survey remained open from February 2–March 4, 2021. All survey procedures were approved by the Columbia University Institutional Review Board (AAAT5271).

### Data analysis

Detailed notes from the expert meeting were synthesized daily by our notetaker and presented to participants at the beginning of the following day of the expert meeting for clarification and feedback. At the end of the consultation meeting, we collected all presentation materials and reviewed them. Using content analysis, we identified key themes and guiding principles from the meeting notes and presentation materials and synthesized them into a final expert meeting report that was reviewed by the consultation committee. Analysis of the online expert survey included preliminary open coding of themes from open-ended questions (SH) and review of codes by another author (MCG). For questions that involved ranking response options, we calculated the median rank across all interviews to estimate the relative priority of responses.

## Results

### Expert consultation meeting

Consultation meeting attendees included 110 participants from 32 countries in Europe (n = 61), Latin America and the Caribbean (n = 4), the Middle East and North Africa (n = 7), North America (n = 10), South Asia (n = 7), South East Asia and the Pacific (n = 7), and sub-Saharan Africa (n = 14). Fifty (45.5%) of participants were female. Professional roles included policymaker or UN/government advisor (48.6%), practitioner (28.6%), researcher (20.0%), or advocate (2.9%) working on issues related to substance use interventions in humanitarian settings. Some participants possess multiple professional roles; however, those reported reflect the role that most closely relates to their primary title and affiliation.

#### Guiding principles for addressing substance use disorders in humanitarian emergencies

Through the content analysis of meeting notes and presentation materials we identified a set of guiding principles for addressing substance use and substance use disorders in humanitarian settings, which included building trust; community engagement, including people with lived experience, families, and caregivers; reducing stigma; ensuring that programs are inter-sectoral, integrated, inclusive, and inter-layered; promoting dignity and empathy; ensuring an ethical ‘do no harm’ approach; and considering culture and context in the design and implementation of substance use disorder treatment programs in humanitarian emergencies.

### Online survey

Thirty-four experts completed an online survey, of whom 11 (32%) were health or low-threshold service providers, 11 (32%) were program planners or administrators, 2 (6%) were policymakers, 1 (3%) was a person with lived experience, and 9 (26%) were researchers/academicians. Of the respondents, 16 (47%) work in humanitarian settings, 4 (12%) work in non-humanitarian settings, and 14 (41%) were not specified.

#### Key gaps in knowledge for addressing substance use disorders in humanitarian emergencies

The online survey participants identified seven key gaps in knowledge regarding the epidemiology and treatment of substance use disorders in humanitarian settings: (1) Substance-use disorder related needs, prevalence of substance use and substance use disorder, and burden of substance use disorder in humanitarian settings (20.6%; e.g. “Under-estimating of the burden of substance use. Many turn a blind eye in camps because they don’t think much about it”); (2) Substance use disorder treatment options and their relative effectiveness and feasibility in humanitarian settings (26.5%; e.g., “lack of awareness of treatment modalities and their efficacy”); (3) The role of context in substance use disorder and implementation of treatment and care services (20.6%; “Understanding the political economy of alcohol availability and consumption”, “Impact of conflict and drug production on the vulnerabilities of young women and girls”); (4) Capacity needed to provide substance use disorder assessment, treatment, and advocacy (44.1%; “Lack of skills in managing different levels of substance use in non-stigmatizing, accessible, community-based settings”); (5) Stigma (20.6%; “Understanding stigma and cultural attitudes towards alcohol use”); (6) Substance use disorder comorbidities (11.8%; “Comorbidity of substance use with other health and mental health concerns”, “Lack of recognition of intersectionality of substance use with critical social issues: IPV, poverty”); and (7) Availability and utilization of substance use services (23.5%; “Evidence-based intervention models”, “Person-centered care”, “Stigma preventing treatment uptake”). Survey participants reported challenges navigating existing operational guidelines to select appropriate interventions, adapting clinical guidelines and implementation approaches in diverse contexts, and generating relevant policy frameworks to inform systems- and policy-level strategies to promote the provision of substance use disorder treatment services for people in need in humanitarian emergencies.

#### Challenges for addressing substance use disorders in humanitarian settings

After describing gaps in knowledge and skills needed to deliver substance use disorder treatments, survey participants were prompted to ascertain the most critical challenges experienced when delivering substance use disorder treatment services in humanitarian settings. At the policy level, both lack of political prioritization and other legal and political constraints were noted as barriers to implementing substance use disorder treatment. The lack of political will, which can be defined as the lack of “committed support among key decision makers for a particular policy solution to a particular problem,” [34] was attributed to the number of competing priorities and was amplified by political resistance to evidence-based substance use treatment approaches (e.g., treatment with long-acting opioid agonists such as methadone/buprenorphine) in some contexts. Criminal justice sanctions for substance use and the lack of awareness among policymakers were considered significant barriers to service delivery.

Challenges related to capacity and knowledge presented obstacles to service delivery. 29% of survey participants mentioned that the lack of skills and resources dedicated to substance use and substance use disorders make it challenging to design and implement treatment interventions. Often, substance use interventions are not addressed due to competing demands on humanitarian agencies and individual provider time. One respondent stated that “support staff like psychiatric nurses and social workers are very difficult to find and there are no training programs to support current models of treatment.” The limited training and capacity were attributed to outdated research and service delivery guidelines. Even in the presence of up-to-date guidance and information, cultural and language barriers present challenges to delivering substance use services in humanitarian emergencies. A consistent theme in the consultation meeting and the online survey related to the uncertainty about how to adapt evidence-based substance use interventions in situations when existing guidance did not fit the needs of a specific population or the resources available in a given humanitarian context.

A central challenge that influenced all other determinants of substance use disorder treatment delivery in humanitarian settings was stigma. People living with a substance use disorder often face social and structural stigma, especially in certain cultural contexts, which can influence how services are implemented and prioritized, help-seeking and utilization of services, and community support. Individuals with substance use disorder displaced by humanitarian emergencies were described as experiencing double-stigma due to substance use disorder and due to being displaced. Gender was described as a factor that modified stigma toward substance use, usually with women experiencing greater stigma relative to men.

#### Priorities for improving the treatment of substance use disorders in humanitarian settings

Online survey participants ranked substance use assessment and intervention activities according to which they thought were most essential (Table 1). The most essential intervention in acute settings included increasing access to services through referrals and linkages to treatment as well as community awareness raising and psychoeducation. In protracted emergencies, increasing access to services was also considered the most essential intervention. Other highly-ranked activities included low-threshold services as well as needs/situational assessments and treatment planning.

**Table 1:** Gaps, challenges, and priorities for addressing substance use disorders in humanitarian settings

Question	Priority	Responses
1. Which interventions do you consider essential to addressing substance use disorder in humanitarian emergencies? [n = 33]	1	Increasing access to services and referrals
	2	Awareness raising and psychoeducation
	3	Low-threshold services
		Needs/situational assessments and treatment planning
2. What information is needed to plan a substance use disorder treatment intervention or program in a humanitarian setting? [n = 31]	1	Substance use patterns in the community
	2	Community norms and attitudes toward substance use
	3	Available services and referral options for substance use services
		Capacity of existing health facilities and staff to provide substance use services
3. For each of the following types of activities, what additional guidance would be most helpful to support the delivery of substance use disorder treatment in humanitarian settings?		
3a. Needs and situational assessment methods [n = 30]	1	Rapid needs and situational assessment
	2	Key informant interviews
	3	Community observations
		Resource mapping
		Surveillance and quantitative survey methodologies
3b. Program planning [n = 29]	1	Service provider training and capacity building
	2	Coordinated care pathways
		Clinical supervision
	3	Accessing to essential medicines
3c. Adaptations of tools and activities [n = 31]	1	Adapted needs assessment and situation analysis tools
		Adapted interventions according to patterns of substance use
		Adapted interventions according to the culture
	2	Adapted case identification and diagnostic tools
	3	Adapted implementation plans to operate within available resources
3d. Interventions [n = 29]	1	Awareness raising and psychoeducation
	2	Low-threshold services
		Evidence-based psychosocial interventions
	3	Evidence-based pharmacological interventions
		Overdose identification and emergency management
3e. Implementation processes [n = 31]	1	Guidance for adapting interventions
		Guidance for implementing interventions
		Guidance on capacity building materials to implement interventions
	2	Links/access to existing clinical guidelines for these interventions
		Development of context-specific guidelines
	3	Brief reference sheets summarizing clinical guidelines
3f. Monitoring and evaluation [n = 24]	1	Designing program objectives and indicators
	2	Selection of means of verification (measures) to assess indicators
	3	Data collection methods for monitoring treatment outcomes

Survey participants indicated that additional guidance is needed to plan for, implement, and evaluate substance use disorder treatment programs in humanitarian settings. In the planning phase, survey respondents reported that knowledge about the epidemiology of substance use disorder in the community is critical to preparing relevant programs (Additional File 1). Participants requested more guidance on how to conduct rapid needs assessments, focus group discussions, key informant interviews, and observations during the early stages of program planning. Beyond characterizing the epidemiology of substance use disorder in the community, participants described the need for tools to map existing resources for addressing substance use disorder to ensure that these resources are leveraged to promote the sustainability of substance use disorder treatment programs. Survey participants detailed the need for guidance on capacity-building and providing training to increase substance use disorder treatment capacity.

Given the variation across humanitarian contexts, online survey participants highly-ranked the development of guidance on the adaptation of interventions to specific populations and settings to improve the implementation of substance use disorder treatment and related services (Additional File 1). More specifically, respondents requested guidance to support for adapting needs assessment and situational analysis tools, adapting interventions according to type and patterns of substance use disorder, and cultural adaptations to improve the relevance of interventions for a given population and setting. Survey respondents reported that new or adapted tools to support awareness-raising and psychoeducation would be most helpful for addressing substance use disorder in humanitarian settings. Five participants stated that they also would benefit from support on how to engage and mobilize community members during program design and implementation. Participants described needing guidance for adapting and implementing interventions and capacity-building materials to implement substance use disorder treatments as well as designing implementation strategies that improve access to care in both acute and protracted emergencies (Additional File 1).

Regarding monitoring and evaluation, survey participants stated the need for tools and information to design program objectives and indicators (Additional File 1). Guidance on the selection of means of verification and data collection methods to monitor treatment outcomes was also prioritized. Detailed results from the online survey rankings are available in Additional File 1.

#### Policy recommendations

The online survey participants were asked to provide policy recommendations for strengthening substance use disorder treatment in humanitarian settings. Four major suggestions were provided to address substance use and substance use disorders in emergency situations. First, increased government support of policies that facilitate access to substance use disorder treatment services was described as a prerequisite to adequately address substance use disorders in emergency settings. Specific recommendations included a health-centered approach instead of criminal justice sanctions for people who use substances and with substance use disorders and allocating more resources to substance use disorder treatment services. Second, participants prioritized health system strengthening and integration of substance use disorder treatment into existing health systems. Substance use disorders often co-occur with other mental and physical disorders, yet remains siloed in delivery. Integration of substance use services into mental health and primary care systems has the potential to improve implementation and decrease stigma. Third, adoption of policies informed by public health was considered an essential strategy for reducing stigma, which was perceived as a mechanism to lower discrimination and increase help-seeking and utilization of substance use disorder treatment. Participants recommended that policy efforts to reduce stigma should be accompanied by community education and awareness raising efforts to increase acceptance of evidence-based drug use disorder treatment interventions, including low-threshold services. Fourth, participants advocated for the inclusion of people who use substances in research on substance use disorder and in the intervention design, development, implementation, and evaluation process to ensure that interventions are culturally acceptable and optimized to benefit the target population. As described by one participant, inclusion of people who use substances and with lived experience in humanitarian settings will lead the target population to “become an asset, not a subject”.

## Discussion

The consultation process consisting of an expert meeting and an online survey revealed key gaps and opportunities for addressing substance use disorders in humanitarian settings. Key gaps in knowledge were directly related to the challenges experienced when implementing substance use disorder interventions in humanitarian settings as well as prioritized needs for future guidance for program planners and service providers. Recommendations to close existing gaps included conducting needs assessments and research to increase our understanding of the epidemiology of substance use disorder in humanitarian settings, improving methods for adapting evidence-based interventions to different populations and contexts, strengthening community- and policy-level awareness, and mainstreaming of substance use disorder treatment in humanitarian settings.

Consistent with previous literature evaluating challenges to implementing substance use interventions in humanitarian settings, aspects of the outer context (e.g., political commitment, policy constraints), human resource capacity, stigma, and limited advocacy for services were central barriers to service delivery [22]. In contrast to previous studies, this research revealed process- and intervention-related implementation challenges within humanitarian settings, such as the need for guidance on adapting assessment tools and operational guidelines to different contexts. Existing substance use disorder screening and assessment tools have yet to be adapted and validated for their use in humanitarian contexts, which is an important part of furthering evidence and knowledge on the epidemiology of substance use disorder in emergency settings. The emphasis on strengthening knowledge that leads to tangible improvements in service delivery resembles the research priorities set for the field of mental health and psychosocial support in humanitarian settings more broadly, which include strengthening knowledge on assessment and epidemiology, adaptation processes, establishing the effectiveness of interventions, and understanding cultural relevance of interventions in humanitarian settings [35].

Recommended adaptations to existing substance use disorder assessment tools and treatment guidelines related more to improving the fit and cultural relevance of the intervention as opposed to adapting implementation processes to operate within available resources, capacities, and type of emergency setting. We anticipated that potentially life-saving interventions, such as overdose management, acute withdrawal management and other interventions to reduce the negative health and social consequences of substance use would be prioritized during acute emergencies while other potentially longer-term psychosocial and pharmacological drug use disorder treatment interventions would be increasingly prioritized in protracted or stabilized settings. Instead, participants considered increasing access to services, raising awareness, and psychoeducation as the most highly prioritized activities in both acute and protracted emergencies. This finding is consistent with many of the minimum health services required to minimize harms related to alcohol and other substance use in emergencies [27].

Several recommendations emerged for strengthening community-based strategies for addressing substance use disorder in humanitarian settings. First, building community awareness and psychoeducation about substance use disorder was described as essential for improving help-seeking and treatment utilization, which is consistent with prior research among conflict-affected and displaced populations [23, 36, 37]. The need for community awareness also related to reducing stigma across multiple levels—community members, providers, policymakers—which often impacted acceptability of substance use interventions, allocation of resources, and political prioritization [38]. Qualitative research conducted among displaced populations identified stigma and norms as major barriers to seeking professional services for substance use disorder [39, 40]. The inclusion of people with lived experience with a substance use disorder in the design and implementation of programs to address substance use disorder in humanitarian settings is an important strategy for mitigating implementation challenges and unintended harm, often related to the consequences of social and structural stigma [41].

Implementation priorities, such as the need to increase access to services and health systems strengthening, reflects the preference among refugee participants for coordinated and integrated care models. Intersectionality and the role of social and structural determinants of health in substance use disorder in humanitarian settings emerged as an important gap in existing knowledge that is consistent with prior qualitative research with populations affected by humanitarian emergencies [39, 42]. Overcoming challenges related to fragmentation of services, particularly for substance use disorder which is often also excluded from mental health systems of care, requires deliberate efforts to understand community demand and engagement in services, map existing resources and referral pathways, identify opportunities for integration of services to address co-occurring social and health problems, and invest in strengthening the capacity of health systems and services, which can be augmented by advocating for greater political commitment to addressing substance use disorders in humanitarian settings.

Through this expert consultation process we identified some key gaps in knowledge, challenges, and priorities for addressing substance use disorders in humanitarian settings. These findings build on existing research documenting current gaps in research and practice within the context of a high and growing burden of substance use disorders globally [43–45], and will contribute to the development of a technical handbook designed to provide specific guidance for the implementation of substance use disorder treatment interventions in humanitarian emergencies. This multi-step consultation process enabled the study investigators to prioritize operational challenges where additional guidance may be helpful to inform program planning and implementation from the perspective of policymakers, program planners, and administrators. Beyond the immediate objective to inform the development of this technical handbook, these results can inform how future investments in addressing substance use disorders in humanitarian settings may be prioritized. These results provide insight into future priorities for research and also areas of opportunity for practice.

A strength of this approach was that the consultation process enabled the investigators to draw from the experience of stakeholders with diverse professional roles (e.g., policymakers, practitioners, researchers) in humanitarian response and/or substance use epidemiology, treatment, and policy. Representation of people with lived experience was limited, which was due, in part, to our recruitment strategy. This research would be strengthened by inclusion of individuals with a history of substance use disorder in a humanitarian setting. We did not collect detailed demographic or other information from participants. However, UNODC operates based on UN rules and regulations and always seeks to have a balance in gender and regional representation in its activities to the extent possible. Few participants possessed expertise in both humanitarian assistance and substance use programming, further highlighting the need to build capacity in this area. Furthermore, recruitment relied on expert nominations from various stakeholders and we did not collect information about individuals who were not selected or declined participation. Our small sample size in the online survey also limits generalizability of these findings or examining differences across world regions, professional role, or other factors. Additionally, the survey was designed by study investigators primarily to inform the development of a technical handbook and, while the survey questions were informed by the key findings of the expert meeting, they were not drawn from standardized or validated measurement tools, which precludes our ability to directly compare our findings to previous research.

## Conclusions

Findings from this study suggest future directions for both research and practice. Critical gaps in knowledge related to the burden and patterns of substance use disorder, the effectiveness of treatment interventions, and the processes for implementing services to address substance use disorders in humanitarian settings are pervasive. Epidemiological, intervention, and implementation research are needed to fill these gaps in knowledge. For practitioners, guidance is needed to translate existing and forthcoming evidence into practice including processes for adaptation, implementation, and evaluation of substance use disorder treatment interventions. Further models are needed to integrate substance use services within mental healthcare and positioned within existing health systems to reduce fragmentation and promote sustainability. In all these future efforts, participation of communities, caregivers, and people with lived experience is needed to reduce the health and social burden caused by substance use disorders and improve the relevance and acceptability of substance use assessments and interventions.

## Supplementary Information

**Additional file 1**. Online survey questions and detailed results.

## Data Availability

The datasets used and analyzed during the current study are available from the corresponding author on reasonable request.

## Abbreviations

UNHCR: United Nations High Commissioner for Refugees
UNODC: United Nations Office on Drugs and Crime
WHO: World Health Organization
