# Supplementary material for: Priorities for addressing substance use disorder in humanitarian settings
Source: Confl Health. 2021 Sep 23;15:71. doi: 10.1186/s13031-021-00407-z (PMC8460183; doi:10.1186/s13031-021-00407-z)
Supplement: Supplementary file 1 — Additional file 1. Online survey questions and detailed results. [file 13031_2021_407_MOESM1_ESM.docx]

**ADDITIONAL FILE 1. ONLINE SURVEY: QUESTIONS AND DETAILED RESULTS**

**SURVEY QUESTIONS**

1. How would you describe your primary professional role?
   1. Policymaker in the health sector
   2. Policymaker in another (non-health) sector
   3. Program planner or manager in a humanitarian setting
   4. Program planner or manager in a non-humanitarian setting
   5. Health service provider in a humanitarian setting
   6. Health service provider in a non-humanitarian setting
   7. Service provider (not in health sector) in a humanitarian setting
   8. Service provider (not in health sector) in a non-humanitarian setting
   9. Person with lived experience with a substance use disorder
   10. Researcher or academician
   11. Other (please specify)
2. Please list (in order of importance, the most important first) the key gaps in knowledge and skills needed to deliver substance use interventions in humanitarian settings.

In this context the word ‘substance’ refers to all psychotropic substances (both illegal and legal) such as cannabis, opioids, amphetamines, hallucinogens, benzodiazepines, alcohol, etc.

With ‘intervention’ we mean activities to reduce the harmful or hazardous use of these substances, including treatment of dependence, intoxication, and withdrawal.

By ‘humanitarian settings’ we refer to a range of situations including natural disasters, conflict, complex political emergencies, and displacement.

1. Humanitarian actors may need guidance when adapting substance use interventions to their local settings. Guidance may focus on different areas. Please rank the need for additional guidance on adaptations required to implement evidence-based substance use interventions in diverse settings from most needed (1) to least needed (8)?
   1. Adapting needs assessment and situation analysis tools
   2. Adapting case identification and diagnostic tools
   3. Adapting interventions according to the type and patterns of substance use
   4. Cultural adaptations to make services more relevant to the target population
   5. Adapting interventions to acute vs. protracted settings
   6. Adjusting program activities to resource limitations
   7. Decision making regarding provision of specialist services vs. training of specialists
   8. Something else (please describe)
2. Describe the most critical challenges in designing and implementing substance use interventions in humanitarian settings. Please be as detailed as possible.
3. Which of the following are interventions that you consider essential to addressing substance use disorder treatment in acute emergencies? Please rank from least essential (1) to most essential (12).
   1. Awareness raising and psychoeducation
   2. Increasing access to services
   3. Low-threshold services (e.g., outreach and drop-in centers, referrals and supporting basic needs, needle/syringe programs, overdose prevention and management, etc.)
   4. Needs/situational assessment and treatment planning
   5. Case identification, screening, and/or diagnosis
   6. Evidence-based psychosocial interventions (e.g., cognitive behavioral therapy, contingency management, motivational interviewing)
   7. Evidence-based pharmacological interventions (e.g., methadone, buprenorphine, naltrexone, acamprosate, disulfiram)
   8. Withdrawal management
   9. Overdose identification and emergency management
   10. Treatment of co-occurring psychiatric and physical health conditions (e.g., managing self-harm, depression, anxiety, psychotic disorders, TB, HIV, HCV, chronic pain)
   11. Recovery management
   12. Other (please specify)
4. Which of the following are interventions that you consider essential to addressing substance use disorder treatment in protracted emergencies? Please rank from least essential (1) to most essential (12).
   1. Awareness raising and psychoeducation
   2. Increasing access to services
   3. Low-threshold services (e.g., outreach and drop-in centers, referrals, supporting basic needs, needle/syringe programs, overdose prevention and management, etc.)
   4. Needs/situational assessment and treatment planning
   5. Case identification, screening, and/or diagnosis
   6. Evidence-based psychosocial interventions (e.g., cognitive behavioral therapy, contingency management, motivational interviewing)
   7. Evidence-based pharmacological interventions (e.g., methadone, buprenorphine, naltrexone, acamprosate, disulfiram)
   8. Withdrawal management
   9. Overdose identification and emergency management
   10. Treatment of co-occurring psychiatric and physical health conditions (e.g., managing self-harm, depression, anxiety, psychotic disorders, TB, HIV, HCV, chronic pain)
   11. Recovery management
   12. Other (please specify)
5. What is the most important information required to plan a substance use treatment intervention or program? Please rank these items by most important (1) to least important (6).
   1. Substance use patterns and types in the community
   2. Community norms and attitudes toward substance use
   3. Available services and referral options for substance use services (prevention, treatment, withdrawal management, etc.)
   4. Capacity of existing health facilities and staff to provide substance use services (e.g., provider competency/training, medication availability, etc.)
   5. Community demand for substance use treatment
   6. Other (please specify)
6. Which of the following substance use needs/situational assessment methods would be most useful to include in a handbook for practitioners in humanitarian settings/working with displaced populations? Please rank these items by most important (1) to least important (7).
   1. Rapid needs and situational assessment
   2. Community observations
   3. Resource mapping
   4. Surveillance and quantitative survey methodologies
   5. Key informant interviews
   6. Facility assessments
   7. Other (please specify)
7. Please describe any tools or information that would be most helpful for conducting substance use needs/situational assessments in humanitarian settings.
8. Which of the following aspects of substance use (including drug use) treatment program planning and preparation should be included in a handbook for practitioners in humanitarian settings/working with displaced populations? Please rank these items by most important (1) to least important (5).
   1. Coordinated care pathways
   2. Service provider training and capacity building
   3. Clinical supervision
   4. Access to essential medicines
   5. Other (please specify)
9. Please describe any tools or information that would be most helpful for program planning and preparation of substance use intervention programs in humanitarian settings.
10. For which type of interventions would new or adapted tools and information be most helpful in humanitarian settings? Please rank these items by most helpful (1) to least helpful (9).
    1. Awareness raising and psychoeducation
    2. Low-threshold services
    3. Evidence-based psychosocial interventions
    4. Evidence-based pharmacological interventions
    5. Withdrawal management
    6. Overdose identification and emergency management
    7. Treatment of co-occurring psychiatric and physical health conditions
    8. Recovery management
    9. Other (please specify)
11. What type of information for the above interventions is most needed? Please rank these items by most needed (1) to least needed (7).
    1. Links/access to existing clinical guidelines for these interventions
    2. Development of context-specific guidelines
    3. Brief reference sheets summarizing clinical guidelines
    4. Guidance for adapting interventions
    5. Guidance for implementing interventions
    6. Guidance on capacity building materials to implement above interventions
    7. Other (please specify)
12. Please describe any tools or information that would be most helpful for implementing substance use treatment in humanitarian settings.
13. Which of the following aspects of monitoring and evaluation should be included in a handbook for practitioners in humanitarian contexts/working with displaced populations? Please rank these items by most important (1) to least important (10).
    1. Designing program objectives and indicators (e.g., developing logframes or theories of change)
    2. Selection of means of verification (measures) to assess indicators (e.g., process, output, outcome, impact indicators)
    3. Data collection methods for monitoring treatment demand
    4. Data collection methods for monitoring treatment outcomes (e.g., qualitative vs. quantitative approaches, using program records, treatment outcome monitoring)
    5. Data collection methods for quality assurance/management of treatment services
    6. Data collection methods for assessing client satisfaction
    7. Management of information collection
    8. Analysis of monitoring and evaluation information
    9. Dissemination strategies
    10. Other (please specify)
14. What policy recommendations or considerations do you have to strengthen substance use prevention and treatment in humanitarian settings? Please be as specific as possible.
15. Do you know of any practitioners or policymakers with knowledge and/or experience on substance use disorder management in humanitarian settings who you would recommend we invite to complete this survey? If so, please provide their name and email address.

**RESULTS FROM THE SURVEY**

| **Table 1. Essential activities for addressing substance use disorders in humanitarian settings** | | |
| --- | --- | --- |
|  | **Median Rank** | |
|  | **Acute emergencies** | **Protracted emergencies** |
| Increasing access to services and referrals | 3 | 2 |
| Awareness raising and psychoeducation | 3 | 4 |
| Low-threshold services (e.g., outreach and drop-in centers, referrals and supporting basic needs, needly/syringe programs, overdose prevention and management, etc.) | 4 | 4 |
| Needs/situational assessment and treatment planning | 4 | 4 |
| Case identification, screening, and/or diagnosis | 5 | 5 |
| Evidence-based psychosocial interventions (e.g., cognitive behavioral therapy, contingency management, motivational interviewing) | 6 | 6 |
| Overdose identification and emergency management | 6 | 9 |
| Evidence-based pharmacological interventions (e.g., methadone, buprenorphine, naltrexone, acamprosate, disulfiram) | 7 | 7 |
| Withdrawal management | 8 | 8 |
| Treatment of co-occurring psychiatric and physical health conditions (e.g., managing self-harm, depression, anxiety, psychotic disorders, TB, HIV, HCV, chronic pain) | 8 | 8 |
| Recovery management | 11 | 11 |
| Other - *Acute Emergencies:* Peer support models, increasing financial coverage/insurance; *Protracted Emergencies:* Peer support models, treatment engagement and retention, increasing financial coverage/insurance and inclusion of services within universal health coverage policies/packages of care | 12 | 12 |

| **Table 2. Priorities for substance use disorder treatment program planning** | |
| --- | --- |
| **Information needed to plan a substance use disorder treatment program** | **Median Rank** |
| Substance use patterns in the community | 2 |
| Community norms and attitudes toward substance use | 3 |
| Available services and referral options for substance use services (prevention, treatment, withdrawal management, etc.) | 3 |
| Capacity of existing health facilities and staff to provide substance use services (e.g., provider competency/training, medication availability, etc.) | 3 |
| Community demand for substance use treatment | 4 |
| Other: legal and policy information | 6 |
| **Requested guidance on needs/situational assessment methods** | **Median Rank** |
| Rapid needs and situational assessment | 1 |
| Key informant interviews | 3 |
| Community observations | 4 |
| Resource mapping | 4 |
| Surveillance and quantitative survey methodologies | 4 |
| Facility assessments | 5 |
| **Requested technical guidance for substance use disorder treatment program planning** | **Median Rank** |
| Service provider training and capacity building | 1 |
| Coordinated care pathways | 3 |
| Clinical supervision | 3 |
| Access to essential medicines | 4 |
| Other: Deinstitutionalized care, meaningful participation/participative development, reducing stigma and marginalization, systems-approaches to developing services to avoid parallel programs and maximize integration within general health systems and sustainability | 5 |

| **Table 3. Priorities for adapting substance use disorder assessment, intervention, and implementation tools** | |
| --- | --- |
| **Guidance needed to adapt…** | **Median Rank** |
| Needs assessment and situation analysis tools | 3 |
| Interventions according to patterns of substance use | 3 |
| Interventions according to the culture | 3 |
| Case identification and diagnostic tools | 4 |
| Implementation plans to operate within available resources | 5 |
| Interventions to acute vs. protracted settings | 6 |
| The selection of interventions to be delivered by a specialist vs. trained non-specialists | 6 |
| Something else: Adaptations to increase participation in SUD services | 8 |

| **Table 4. Priorities for substance use disorder treatment implementation** | |
| --- | --- |
| **Priority interventions** | **Median Rank** |
| Awareness raising and psychoeducation | 2 |
| Low-threshold services | 3 |
| Evidence-based psychosocial interventions | 3 |
| Evidence-based pharmacological interventions | 4 |
| Overdose identification and emergency management | 4 |
| Withdrawal management | 5 |
| Treatment of co-occurring psychiatric and physical health conditions | 6 |
| Recovery management | 8 |
| Other: community engagement | 9 |
| **Guidance needed to implement substance use interventions** | **Median Rank** |
| Guidance for adapting interventions | 3 |
| Guidance for implementing interventions | 3 |
| Guidance on capacity building materials to implement above interventions | 3 |
| Links/access to existing clinical guidelines for these interventions | 4 |
| Development of context-specific guidelines | 4 |
| Brief reference sheets summarizing clinical guidelines | 5 |
| Other: triage screening tools like stimulant health checks, guidance on doing community consultations with people who use drugs, quality standards | 7 |

| **Table 5. Priorities for monitoring and evaluation of substance use disorder interventions** | |
| --- | --- |
| **Guidance needed to monitor and evaluate programs** | **Median Rank** |
| Designing program objectives and indicators (e.g., developing logframes or theories of change) | 1 |
| Selection of means of verification (measures) to assess indicators (e.g., process, output, outcome, impact indicators) | 3 |
| Data collection methods for monitoring treatment outcomes (e.g., qualitative vs. quantitative approaches, using program records, treatment outcome monitoring) | 3.5 |
| Data collection methods for quality assurance/management of treatment services | 4 |
| Data collection methods for monitoring treatment demand | 5 |
| Data collection methods for assessing client satisfaction | 6 |
| Management of information collection | 6 |
| Analysis of monitoring and evaluation information | 8 |
| Dissemination strategies | 9 |
| Other: Peer audit and models for capturing peer feedback about the quality and accessibility of services | 10 |
